# Supplementary material for: Elliptical Bloch skyrmion chiral twins in an antiskyrmion system
Source: Nat Commun. 2020 Feb 28;11:1115. doi: 10.1038/s41467-020-14925-6 (PMC7048809; doi:10.1038/s41467-020-14925-6)
Supplement: Supplementary file 1 — Supplementary Information [file 41467_2020_14925_MOESM1_ESM.pdf]

Supplementary Information

Elliptical Bloch skyrmion chiral twins in an antiskyrmion system

Jagannath Jena[1#], Börge Göbel[1,2#], Tianping Ma[1], Vivek Kumar[3], Rana Saha[1], Ingrid Mertig[2,1], Claudia Felser[3], and Stuart S. P. Parkin[1*]

[1] Max Planck Institute of Microstructure Physics, Weinberg 2, 06120 Halle (Saale), Germany

[2] Institute of Physics, Martin Luther University Halle-Wittenberg, 06120 Halle (Saale), Germany

[3] Max Planck Institute for Chemical Physics of Solids, Nöthnitzer Strasse 40, 01187 Dresden, Germany

*email: stuart.parkin@mpi-halle.mpg.de

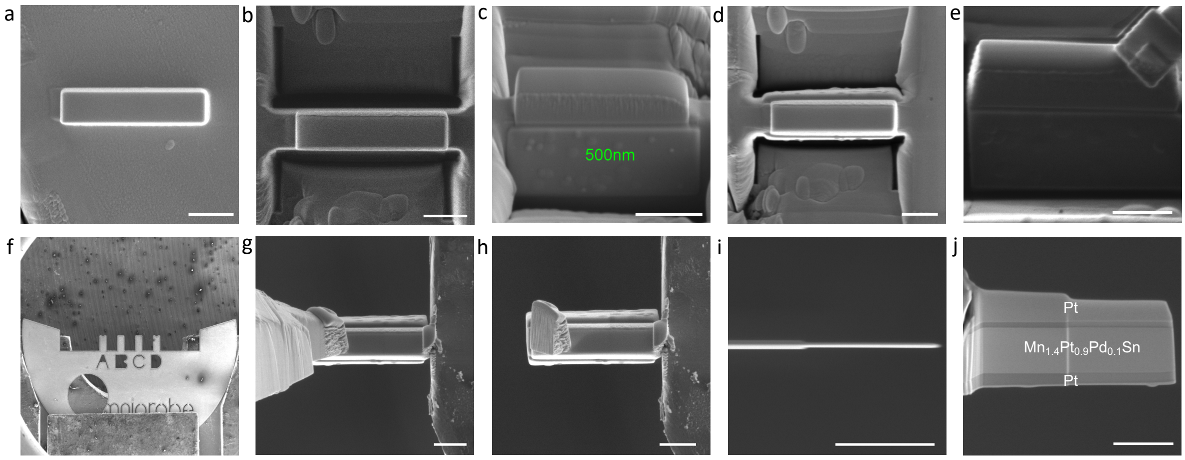

Supplementary Figure 1 | Lamella preparation in a focused ion beam. The ten panels show chronologically how the thin lamella of $Mn_{1.4}Pt_{0.9}Pd_{0.1}Sn$ **j** is prepared from the initial [001] oriented grain shown in **a**. The procedure is explained in detail in the main text of this Supplementary information. The 500 nm mark in **c** is given for the approximate thickness of Pt deposited on one side of the surface. Scale bars in each image correspond to 3 μm.

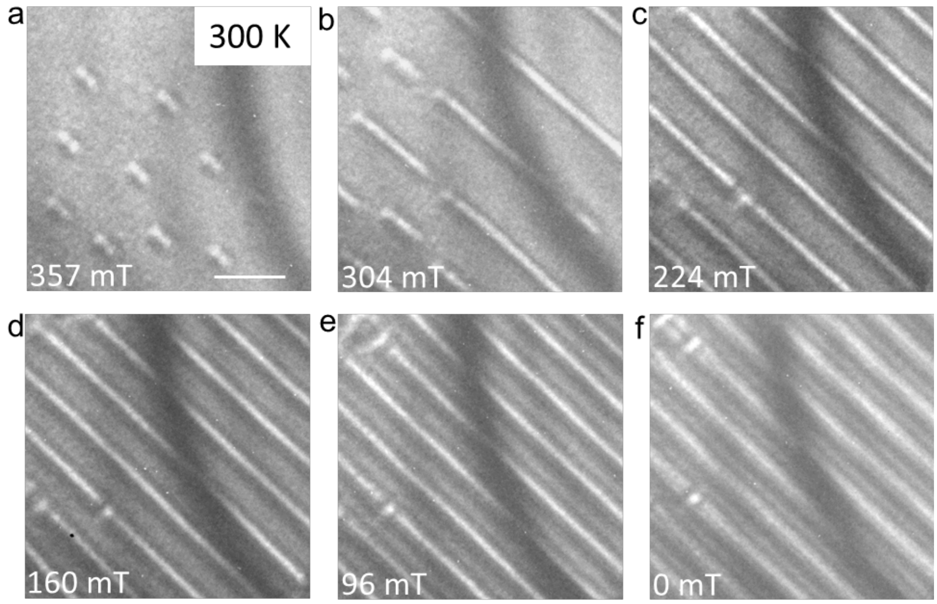

Supplementary Figure 2 | Field dependence of a sparse array of individual antiskyrmions. Before taking the LTEM image shown in **a**, the sample has been temporarily tilted to provide an in-plane magnetic field that helps stabilizing the sparse array of antiskyrmions. In **b** - **f** the magnetic field remains along the pole direction. It is gradually decreased, as indicated. The scale bar corresponds to 300 nm.

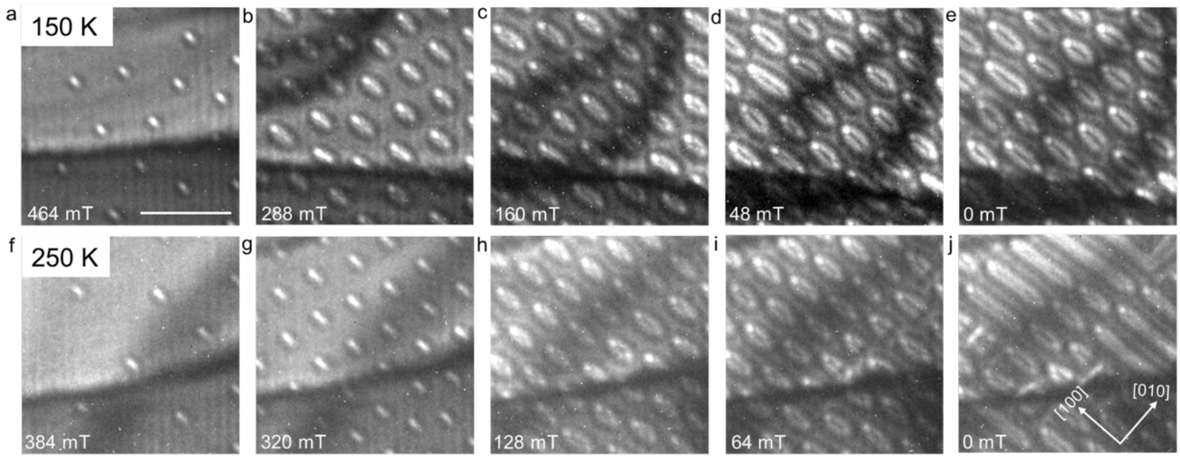

Supplementary Figure 3 | Field stabilized elliptical-skyrmions at 150K and 250K. The presented image sequences are by analogy with Fig. 3 of the main text but for different temperatures. Figures **a-e** show the observations at 150K, while **f-j** have been taken at 250K. The scale bar corresponds to 500 nm.

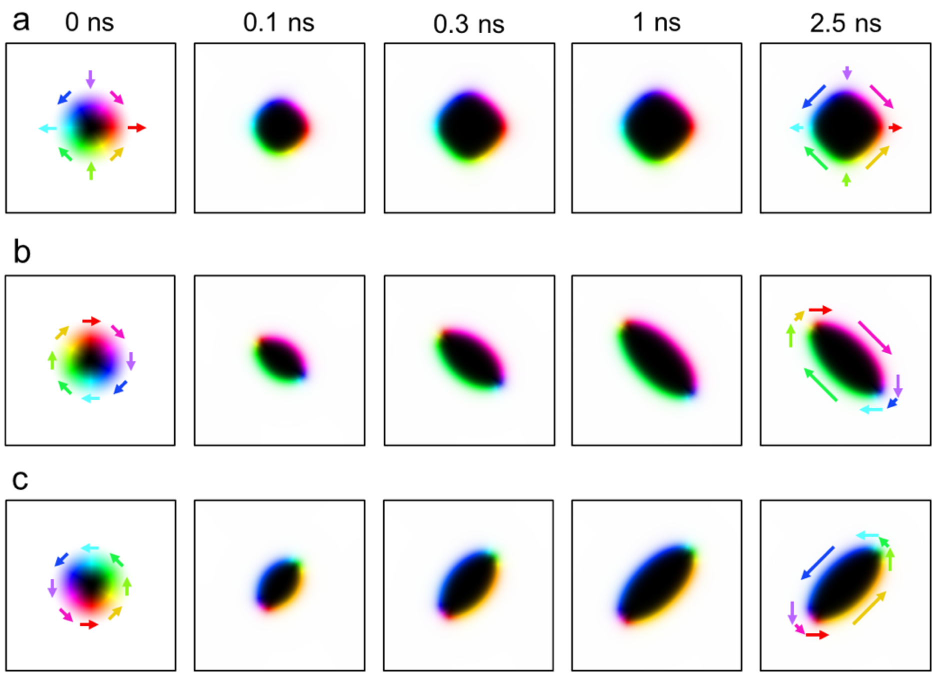

Supplementary Figure 4 | Simulated relaxation of spin textures. The relaxation of different circular spin textures (left column) is shown. In **a**, the relaxation of a circular antiskyrmion to a square-shaped antiskyrmion is shown. **b** and **c** show the relaxation of two Bloch skyrmions with opposite chiralities to two differently elongated elliptical-skyrmions. The arrows and the colors schematically visualize the in-plane magnetization. The right panels correspond to Figs. 5d, 5b and 5c, respectively.

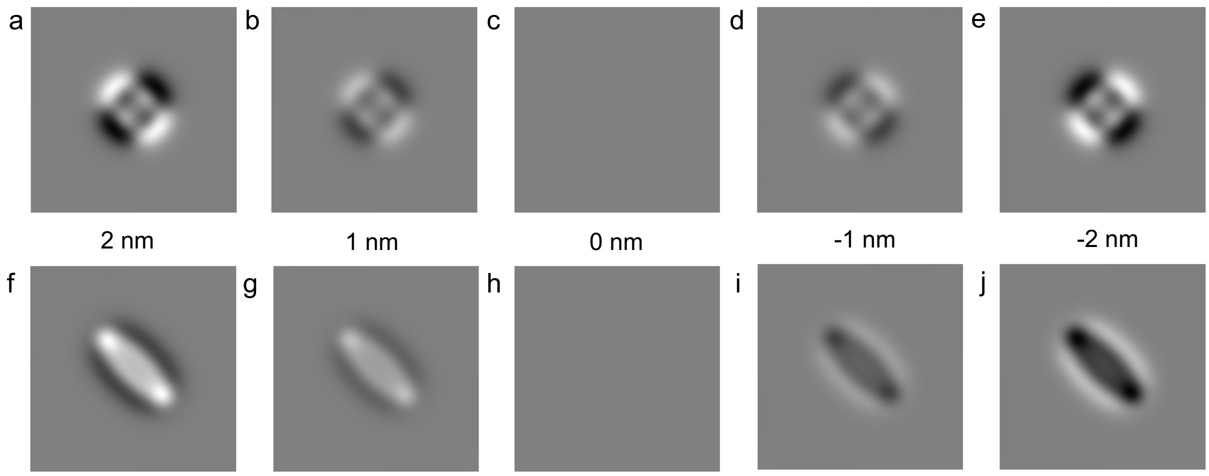

Supplementary Figure 5 | Simulated LTEM contrast for different defocus values. In **a-e** the square-shaped antiskyrmion is shown and in **f-j** the elliptical skyrmion is shown. The values of the defocus parameter (see methods section in the paper) are indicated. Panels **a** and **f** correspond to the figures shown in Fig. 5 of the paper.

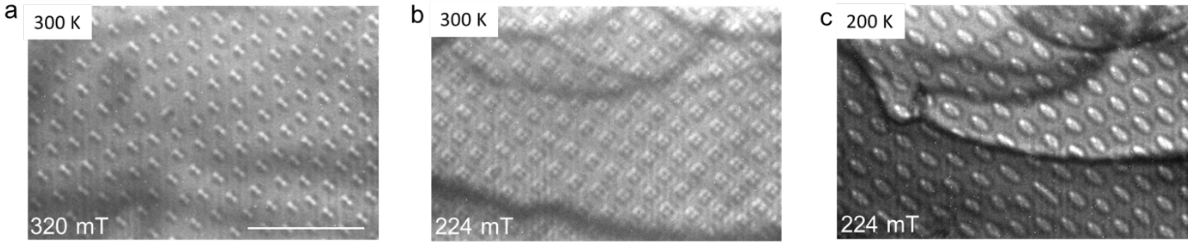

Supplementary Figure 6 | Comparison of lattices of antiskyrmions and elliptical-skyrmions. In **a** and **b**, arrays of round and square-shaped antiskyrmions are shown, respectively. These results correspond to Fig. 1 from the paper. In **c** an elliptical-skyrmion crystal is shown at 200 K, similar to Fig. 3 from the paper. The scale bar corresponds to 1 μm.

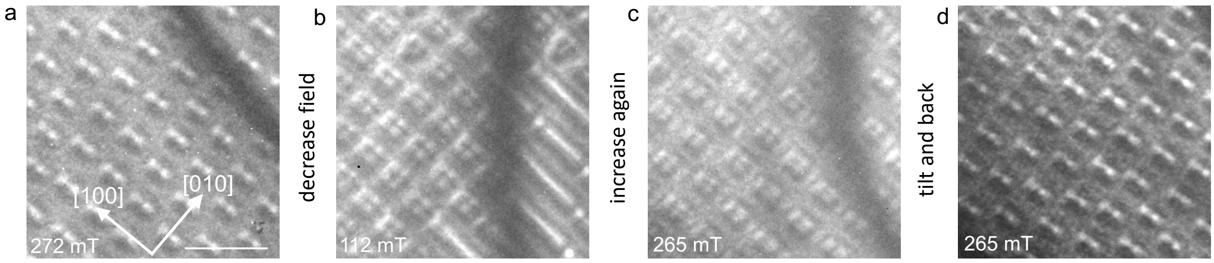

Supplementary Figure 7 | Switching between round and square shaped antiskyrmions. In **a**, a lattice of round antiskyrmions is stabilized for a magnetic field applied along the [001] zone axis after temporarily applying an in-plane component of the field by tilting the sample. Afterwards, the magnetic field is decreased and the texture transforms to a square-shaped antiskyrmion lattice shown in **b**. In **c** the field is increased again but the texture remains a lattice of square-shaped antiskyrmions. Only after temporarily providing an in-plane field, the configuration turns back to the lattice of round antiskyrmions, shown in **d**. The scale bar corresponds to 400 nm.

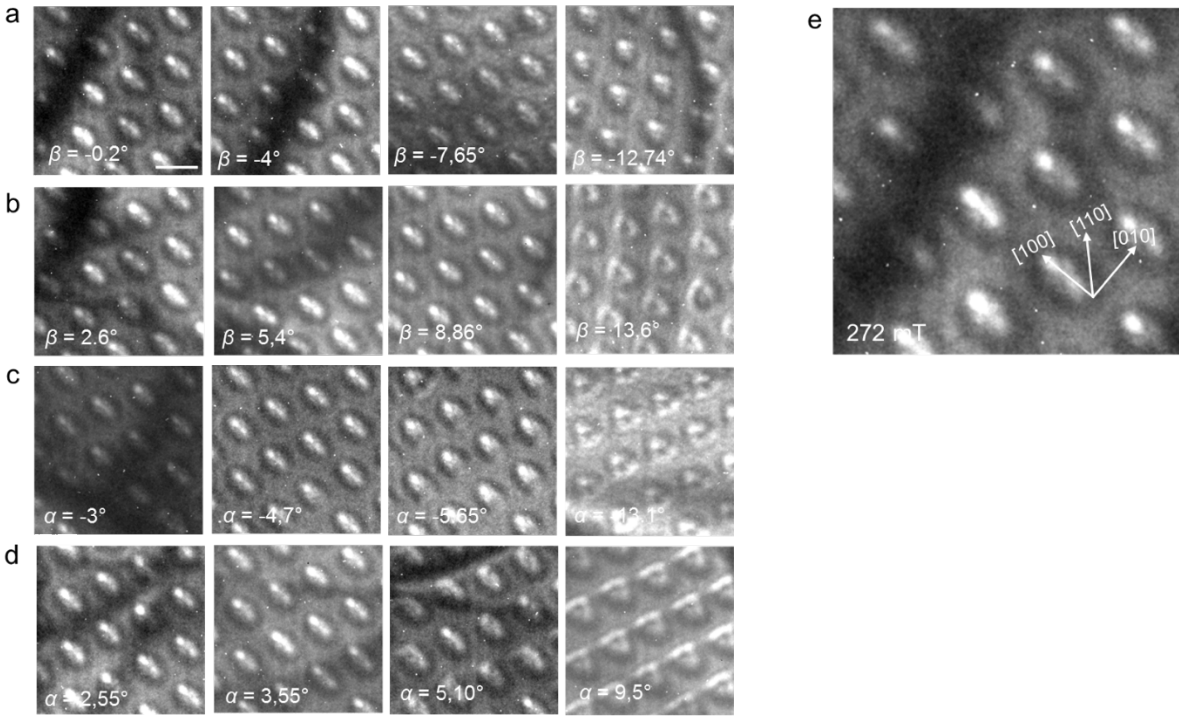

Supplementary figure 8 | Field-tilting effect on elliptical-skyrmions. In **a**, **b**, **c**, and **d** we start from a lattice of elliptical-skyrmions stabilized at 150 K and 272 mT and tilt the sample such the field is effectively tilted along the angles α and β that correspond to the different {11} directions. In all cases, the texture deforms to a triangular shape but after returning to a perpendicular field, the configurations turn back to the initial elliptical-skyrmion phase shown in **e**. The scale bar corresponds to 100 nm.

Supplementary Note 1. Preparation of a thin lamella for the Lorentz TEM investigation

In Supplementary Figure 1 we show how we prepared the surface parallel thin lamella of $Mn_{1.4}Pt_{0.9}Pd_{0.1}Sn$. The sample was prepared in a Ga^+ focused ion beam (FIB) operating at an acceleration voltage of 30 keV [FEI, Nova nano Lab]. The ion beam column is 52° with respect to the electron beam column.

First, we deposit Pt via a GIS (Gas Injection System) on the top of the [001] oriented grain by electron and ion beam techniques (Supplementary Figure 1a). Then we remove a few micrometers from the outside region of Pt using ion beams with a higher current (Supplementary Figure 1b). Thereafter we start to polish both sides of the lamella to remove the damaged portions of the surface using the ion beam at lower currents. The final polishing has been conducted at an energy of 2 keV. At this point the lamella has a thickness of nearly 3μm.

In the next step, in a tilted condition (52° degree with respect to the electron column), we deposited Pt of a few nanometers thickness on both sides of the thicker lamella using an electron beam (Supplementary Figure 1c). The steps of smooth polishing and Pt deposition are required here, because when we lift out the lamella, our lamella must not be damaged by the ion beam (during the following cutting process of the lamella for the process of lifting out, its surface is perpendicular to ion beam). Supplementary Figure 1d shows the deposition of Pt on both sides of the lamella.

To lift out the lamella, first we cut it in an 'L' shape so that one side of the lamella is attached to the bulk sample and the other two sides will be cut by the ion beam. We then attach a nano-manipulator to the sample by depositing Pt (Supplementary Figure 1e) and cut out the remaining part that is attached to the bulk sample. We attach our thicker lamella to a four-arm grid, which is seated perpendicularly to the electron column (Supplementary Figure 1f). The lamella is

attached to any of the four arms and a few nanometers of Pt are deposited on the junction between the grid and the sample (Supplementary Figure 1g).

To separate out the lamella from the attached nanomanipulator, we cut the end portion of it with an ion beam (Supplementary Figure 1h). Then we vent out the whole FIB stem and again place the four-arm grid parallel to electron column. After this we tilted the sample by 52°. More Pt is deposited on the surface of the lamella to protect the lamella from the ion beam. Then we thin down both sides of the lamella by cleaning the cross section pattern with lower currents to a few nanometers. The final polishing is done at an energy of 2 keV to remove surface amorphization.

The final film thickness is ∼170nm (Supplementary Figure 1i). The surface of the lamella is shown in Supplementary Figure 1j. The left portion has a larger thickness of approximately 400nm and the right part is the portion that has been investigated in the present study.

Supplementary Note 2. Field dependence of a sparse array of individual antiskyrmions

In Fig. 1g-k we have shown that the explained tilted-field protocol allows for the formation of a dense array of antiskyrmions that survives even at zero field. In Supplementary Figure 2 we show how a sparse array of antiskyrmions behaves when the field is decreased without providing an in-plane component. In Supplementary Figure 2f, zero field is reached and the antiskyrmions have disappeared. In contrast to Fig. 1k the system is now in a helical phase. This shows once again that the observed magnetic textures are metastable and that the type of the stabilized texture strongly depends on the experimental protocol. Two (topologically) distinct spin textures can be metastabilized under the same temperature and magnetic field.

Supplementary Note 3. Observation of elliptical-skyrmions at 150 K and 250 K

In Supplementary Figure 3 we present LTEM images of elliptical-skyrmions at the temperatures 150K and 250K to compliment the presented images for 200K from the main text. We follow the same protocol in each case and observe a similar trend for the metastabilization of elliptical-skyrmions each time.

The only essential difference is that at the highest temperature of 250K the elliptical-skyrmion lattice transitions to the helical state at zero magnetic field (Supplementary Figure 3j), while the lattice is rather periodic at 200K (Fig. 3e of the main text) and 150K (Supplementary Figure 3e). A possible reason is that the strength of the dipole-dipole interaction – the stabilizing mechanism of skyrmionic textures in the present material – is decreased, since the net magnetization decreases with increasing temperature[1]. At the next higher temperature (300K presented in Fig. 1 of the main text) no elliptical-skyrmions have been observed when the presented experimental protocol was used indicating that the DMI has become more important than the dipole-dipole interaction at this temperature.

Supplementary Note 4. Field-tilting effect on elliptical-skyrmions

To investigate if the chirality and the elongation axis of the elliptical-skyrmions is determined by the direction of the in-plane field, we varied the effective tilting direction of the field along different {11} directions (indicated by the angles α and β in Supplementary Figure 8). Starting from the elliptical-skyrmion lattice shown in Supplementary Figure 8e, tilting along the specified direction and coming back to the pole direction, the magnetic texture always returns to the same configuration. Therefore, in our sample under the presented protocol, the skyrmion chirality is not determined by the in-plane tilting direction.

Supplementary References

1. Nayak, A. K. et al. Magnetic antiskyrmions above room temperature in tetragonal Heusler materials. *Nature* **548**, 561 (2017).
